# Identification of the terebrantian thrips (Insecta, Thysanoptera) associated with cultivated plants in Java, Indonesia

**DOI:** 10.3897/zookeys.306.5455

**Published:** 2013-06-03

**Authors:** Dewi Sartiami, Laurence A. Mound

**Affiliations:** 1 Department of Plant Protection, Bogor Agicultural University, Bogor, Indonesia; 2 CSIRO Ecosystem Sciences, PO Box 1700, ACT 2601, Australia

**Keywords:** Identification keys, Indonesia, Thripidae, cultivated plants

## Abstract

An illustrated identification key is provided to 49 species of Thysanoptera, Terebrantia that have been found in association with cultivated plants in Java. This is the first published identification system to this group of insects from Indonesia, and includes 15 species not previously recorded from Indonesia, and a further three species not previously recorded from Java. A table is provided indicating the plants from which thrips were taken.

## Introduction

Judging from a list published by zur Strassen (1994), the Thysanoptera fauna of Indonesia appears to be well known. That list documents 433 thrips species from Indonesia, 279 of which are from Java. Although a useful resource for checking the names and presence of a genus or species, the list has limitations as a basis for further work by Indonesian entomologists. The alphabetic arrangement of genera, rather than grouping by sub-order and family, makes it difficult to gauge the biological diversity of the fauna, particularly as biologies are not indicated, and no advice given on identification methods. For the Indonesian thrips fauna no practical identification system exists, although some progress has been made recently in studies on the thrips fauna of Peninsular Malaysia (Mound and Azidah 2009; Mound and Ng 2009; Mound et al. 2012), and a key to the Asian species of the genus *Thrips* was provided by Palmer (1992). However, many of the Asian species treated in these keys remain known only from one or a few specimens. There is thus no knowledge of the variability of such species, and their validity remains questionable (Mound 2005). Extensive field studies are needed, to establish the structural variation within and between populations of several species, before a truly robust identification system can be produced.

Most of the species listed from Indonesia by zur Strassen (1994) were collected or acquired in the 1920’s by H.H.Karny whilst entomologist at the Buitenzorg Zoological Museum (Bogor). They were described either by Karny himself, or subsequently by H. Priesner who inherited most of Karny’s slide collection. These slides are now housed at the Senckenberg Museum, Frankfurt. Unfortunately, Karny considered that the mouth parts of thrips would be found to hold useful taxonomic characters. Thus he slide-mounted most specimens ventral side uppermost, and as a result it is often difficult or impossible to study the dorsal character states that are now considered essential. Moreover, a high proportion of the specimens are damaged or distorted.

The 279 species of Thysanoptera listed by zur Strassen (1994) from Java are predominantly members of the sub-order Tubulifera, family Phlaeothripidae. In contrast, most thrips that are of importance to agriculture and horticulture are members of the second sub-order, the Terebrantia, and only 90 of these are listed from Java, of which 23 are members of the genus *Thrips*. For Indonesian entomologists, the absence of suitable identification keys, also the absence of an authentic reference collection, makes the task of studying these small insects particularly difficult. The objective of the present work is to provide a means of identification of the species associated with cultivated plants in Java. The intention is to enable economic entomologists in Indonesia to identify the pest thrips associated with their crops, and thus facilitate studies on the biology of these insects. In this way it is hoped that an understanding of the rich and complex Indonesian thrips fauna may be developed progressively.

The material discussed here has been collected at the following sites: West Java at Bogor and Purwakarta (lowland sites), and at Bandung and Cianjur (highland sites); Central Java at Semarang and Demak (lowland), and Banjarnegara, Tegal (highland); East Java at Kediri, Situbondo and Malang (lowland). In the text below, *indicates the three species that are newly recorded from Java, and **indicates 15 species not previously recorded from any of the Indonesian islands. The slide-mounted specimens on which this report is based are housed in the Bogor Agricultural University. It is particularly noteworthy that the worldwide pest, Western Flower Thrips, *Frankliniella occidentalis*, has not yet been recorded from anywhere in Indonesia.

The classification adopted here is the widely accepted one that is web available (ThripsWiki 2013). That web site provides full nomenclatural details of all taxa discussed in this paper, together with references and other details. Moreover, many of the common widespread species that are considered here are fully described and illustrated in the web-available identification systems for Australia and California (Mound et al. 2012; Hoddle et al. 2012). Another useful source of information is the key to Thripinae genera associated with plant quarantine in Japan (Masumoto 2010).

## Key to species

**Table:** 

1	Antennae with 9-segments; sensoria on segments III–IV linear, either longitudinal along the segments or transverse; wing apex rounded (Fig. 2)	[Aeolothripidae] 2
–	Antennae usually with 7 or 8 segments (rarely with 6 or 9); sensoria on segments III–IV emergent, either forked or simple; wing apex more or less pointed (Fig. 6)	[Thripidae] 3
2	Antennal segments III–IV long, III is 9 times as long as width (Fig. 1); segments I–II white	*Franklinothrips vespiformis*
–	Antennal segments III–IV scarcely longer than wide (Fig. 2), dark brown with many long setae	*Mymarothrips bicolor*
3	Hind tarsi more than 0.5 as long as hind tibia	*Pseudodendrothrips* sp.
–	Hind tarsi less than 0.3 as long as hind tibia	4
4	Fore wing anterior margin with cilia arising ventrally behind the margin (Fig. 5); metathoracic furca Y-shaped and extending to mesothorax (Fig. 8) [body white, brown area between eyes; fore wing banded]	*Asprothrips navsariensis*
–	Fore wing anterior margin with cilia arising at margin; metathoracic furca usually not as above	5
5	Antennal segment IV with parallel-sided apical neck that is 50% as long as this segment (Fig. 4); head and body yellow with no dark pigment	*Zaniothrips ricini*
–	Antennal segment IV without elongate apical neck (Fig. 3), body colour various	6
6	Fore wing dark and setae on costa and first vein long with apices capitate (Fig. 38), second vein with no setae; head and pronotum strongly reticulate, reticles with internal markings	*Echinothrips americanus*
–	Fore wing setae not capitate, second vein rarely without setae; reticulation on head and thorax rarely with any internal markings	7
7	Head, body and fore femora boldly reticulate (Figs 13, 14); mesothoracic furca without a median spinula; fore wing first vein usually fused to costa	8
–	Head and body not boldly reticulate, fore femora without reticulation; mesothoracic furca usually with a median spinula (Fig. 9); fore wing first vein distinct from costa	15
8	Abdominal tergite II anterolaterally with paired areas of specialised sculpture, either prominent recurved claw-like microtrichia or closely spaced ridges (Figs 11, 12)	9
–	Abdominal tergite II without paired areas of specialised sculpture	12
9	Dorsal surface of head with prominent transverse ridge (cf. Fig. 14)	10
–	Dorsal surface of head without a transverse ridge	11
10	Fore wing costal setae longer than costal cilia (Fig. 10); tergite II anterolaterally with paired area of recurved claw-like microtrichia (Fig. 12)	*Copidothrips octarticulatus*
–	Fore wing costal setae shorter than costal cilia; tergite II anterolaterally with paired areas of parallel ridges (Fig. 11)	*Panchaetothrips indicus*
11	Terminal setae on tergite X pointed	*Astrothrips* sp.
–	Terminal setae on tergite X with apices expanded	*Elixothrips brevisetis*
12	Fore wing uniformly dark, with two rows of prominent setae	*Selenothrips rubrocintus*
–	Fore wing pale or banded, without two rows of large setae	13
13	Fore wing second vein with row of setae (Fig. 6); tergal antecostal ridge forming series of arches (Fig. 15)	*Helionothrips ananthakhrisnani*
–	Fore wing with no major setae; tergites without arch-like sculpture	14
14	Head with equiangular reticulation, without transverse ridge (Fig. 13)	*Heliothrips haemorrhoidalis*
–	Head with complex irregular sculpture and transverse ridge (Fig. 14)	*Rhipiphorothrips pulchellus*
15	Head strongly prolonged in front of eyes (Fig. 18)	*Organothrips indicus*
–	Head rarely extending in front of eyes	16
16	Lateral areas of abdominal tergites II–VII with numerous large microtrichia on broad triangular bases (Fig. 16); fore wing banded, without setae	*Dendrothripoides innoxius*
–	Lateral thirds of tergites without such large microtrichia; fore wing veins with setae	17
17	Pronotum with no long setae	18
–	Pronotum with at least 1 pair of prominent setae	19
18	Body sharply bicoloured, brown with abdominal segments III–V yellow; metanotum irregularly reticulate	*Anaphothrips sudanensis*
–	Body uniformly dark brown; metanotal sculpture transverse (Fig. 19)	*Dichromothrips corbetii*
19	Tergites with many closely spaced rows of fine microtrichia laterally (Fig. 17)	*Scirtothrips dorsalis*
–	Tergites without closely spaced rows of fine microtrichia laterally (Fig. 26)	20
20	Abdominal tergites without paired ctenidia laterally	21
–	Abdominal tergites V–VIII with paired ctenidia laterally (Figs 26, 27)	30
21	Pronotum with 6 pairs of long setae (Fig. 39)	*Scolothrips rhagebianus*
–	Pronotum with no more than 3 pairs of long setae	22
22	Tergite VIII posterior margin with neither craspedum nor comb (Fig. 25)	*Bathrips melanicornis*
–	Tergite VIII posterior margin with either a comb of microtrichia or a craspedum	23
23	Tergite VIII posterior margin with a craspedum but no comb (Fig. 7)	24
–	Tergite VIII posterior margin with a comb of microtrichia (Fig. 26), either complete or present only laterally	25
24	Sternite VII with three pairs of marginal setae equidistant from each other	*Craspedothrips minor*
–	Sternite VII posteromarginal setae S1 and S2 close together medially, and distant from lateral setal pair S3	*Rhamphothrips* sp.
25	Pronotum anterior margin with 1 pair of elongate setae (Fig. 20); metanotum almost without sculpture medially; abdominal tergites III–VI strongly reticulate (Fig. 21)	*Ayyaria chaetophora*
–	Pronotum anterior margin with no long setae; metanotum sculptured medially; tergites not strongly reticulate	26
26	Metanotum with equiangular reticulation (Fig. 23); ocellar setae pair III shorter than distance between two ocelli (Fig. 22); antennal segment I without pair of dorso-apical setae	*Lefroyothrips lefroyi*
–	Metanotal reticulation not equiangular; ocellar setae pair III longer than distance between two ocelli; antennal segment I with pair of setae at dorsal apex (Fig. 3)	27
27	Female tergite VIII with comb of long microtrichia complete medially	28
–	Female tergite VIII posterior margin with a few microtrichia laterall but none medially	29
28	Meso and metathroacic furca with long spinula; ocellar setae pair I arising side-by-side; fore wing pale or with transverse darker bands	*Mycterothrips* sp.
–	No spinula on metathoracic furca (cf. Fig. 9); ocellar setae pair I arising one setae in front of the other; fore wing uniformly dark	*Ceratothripoides brunneus*
29	Abdominal sternite VII posteromarginal setae all arise on margin (Fig. 24); antennal segments IV and V yellow on basal half	*Megalurothrips typicus*
–	Median pair of marginal setae on abdominal sternite VII arise in front of margin	30
30	All antennal segments brown	*Megalurothrips distalis*
–	Antennal segment III yellow	*Megalurothrips usitatus*
31	Ctenidia on tergite VIII anterolateral to spiracle (Fig. 26); head with pair of setae in front of first ocellus	32
–	Ctenidia on tergite VIII posteromesad to spiracle (Fig. 27); head without pair of setae in front of first ocellus	33
32	Tergite VIII posterior margin with complete comb of microtrichia; ocellar seta III arise on anterior margin of ocellar triangle	*Frankliniella intonsa*
–	Tergite VIII posterior margin with no comb of microtrichia; ocellar seta III arise between posterior pair of ocelli	*Frankliniella schultzei*
33	Abdominal sternites IV–VI with discal setae (Fig. 35)	34
–	Abdominal sternites IV–VI with setae only at posterior margin (cf Fig. 24)	44
34	Tergites II–VIII posterior margin with toothed craspedum (Fig. 27); prosternum with 2 or more pairs of setae	*Microcephalothrips abdominalis*
–	Tergites II–VIII without a toothed marginal craspedum; prosternum with no setae	35
35	Antennal segments III and IV with sensorium simple	*Bolacothrips striatopennatus*
–	Antennal segments III and IV with sensorium forked	36
36	Pronotum with only one pair of long posteroangular setae	*Thrips unipinus*
–	Pronotum with two pairs of prominent posteroangular setae	37
37	Sternite VII without discal setae (Fig. 35); fore wing first vein setal row complete; pleurotergites with discal setae	*Thrips parvispinus*
–	Sternite VII with discal setae present; fore wing first vein setal row interrupted; pleurotergites usually with no discal setae	38
38	Pronotal posteroangular setae short, usually shorter than median metanotal setae	*Thrips melastomae*
–	Pronotal posteroangular setae at least as long as median metanotal setae and usually longer	39
39	Pleurotergites with discal seta (Fig. 30); metanotum without campaniform sensilla	*Thrips alliorum*
–	Pleurotergites without discal seta (Fig. 32); metanotum usually with paired campaniform sensilla	40
40	Abdominal tergite II with 4 setae laterally	41
–	Abdominal tergite II with 3 setae laterally	43
41	Metanotal median setae arising just behind anterior margin; body colour varying from yellow to brown, but usually with tergite X brown and dark markings medially on some tergites	*Thrips coloratus*
–	Metanotal median setae arising at anterior margin; body brown, head and thorax sometimes yellow	42
42	Postocular setae pair II minute; mesonotum with no sculpture close to anterior pair of campaniform sensilla (Fig. 36); fore wing clavus with terminal seta shorter than sub-terminal seta	*Thrips florum*
–	Postocular setae pair II at least half as long as setae I and III; mesonotum with sculpture lines close to anterior pair of campaniform sensilla (Fig. 37); fore wing clavus with terminal seta longer than sub-terminal seta	*Thrips hawaiiensis*
43	Antennae with 8 segments; fore wing first vein with 7 setae on distal half; metanotum reticulate with markings inside the reticles (Fig. 34)	*Thrips simplex*
–	Antennae with 7 segments; fore wing first vein with 3 setae on distal half; metanotal reticulations without internal markings	*Thrips safrus*
44	Head with ocellar setae pair II longer than pair III (Fig. 28) [fore wing uniformly brown]	*Stenchaetothrips biformis*
–	Head with ocellar setae pair II shorter than pair III	45
45	Abdominal tergites III–V with median pair of setae more 0.5 as long as the median length of their tergite (Fig. 29)	*Thrips nigropilosus*
–	Abdominal tergites III–V with median pair of setae scarcely 0.3 as long as tergite	46
46	Fore wing first vein with setal row almost complete	*Thrips malloti*
–	Fore wing first vein with wide gaps distally in setal row	47
47	Pleurotergites with closely spaced rows of fine microtrichia (Fig. 32); body variable in colour but ocellar pigment grey and never red	*Thrips tabaci*
–	Pleurotergites without such rows of microtrichia; ocellar pigment red	48
48	Tergite VIII posterior margin with no comb medially; body dark brown	*Thrips javanicus*
–	Tergite VIII posterior margin with complete comb medially; body yellow	*Thrips palmi*

**Figures 1–9.**
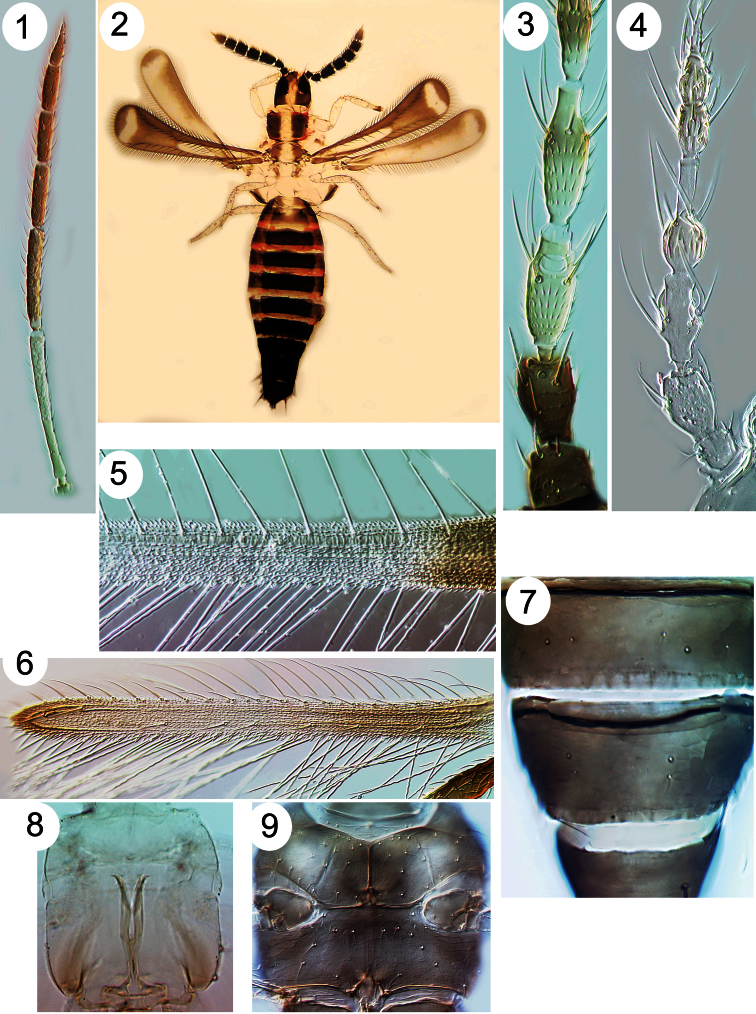
Thripinae from Java. **1** *Franklinothrips vespiformis*, antenna **2** *Mymarothrips bicolour*, female **3** *Megalurothrips typicus* antennal segments I–V 4 *Zaniothrips ricini* antenna **5** *Asprothrips navsariensis*, fore wing **6** *Helionothrips ananthakrishnani* fore wing **7** *Craspedothrips minor*, tergites VII & VIII **8** *Asprothrips*, metafurca **9** *Echinothrips americanus*, with mesosternal spinula.

**Figures 10–18.**
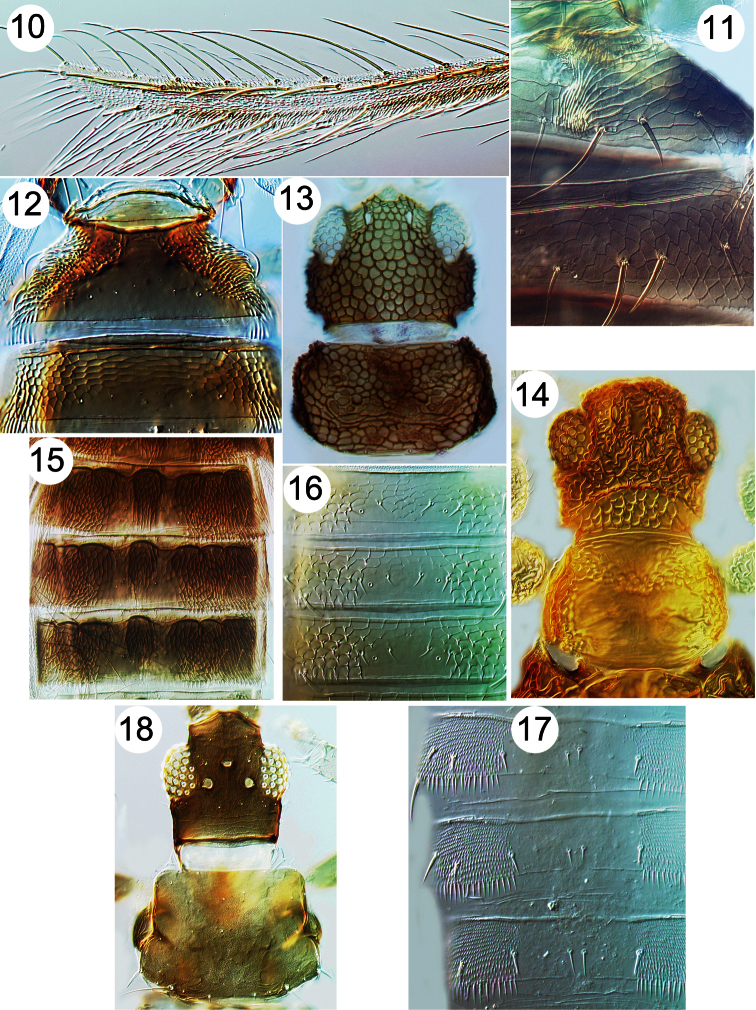
Thripinae from Java. **10** *Copidothrips octarticulatus* fore wing **11** *Panchaetothrips indicus*, tergites I & II **12** *Copidothrips octarticulatus*, tergites I & II **13** *Heliothrips haemorroidalis*, head & pronotum **14** *Rhipiphorothrips pulchellus*, head & pronotum **15** *Helionothrips ananthakrishnani*, tergites **16** *Dendrothripoides innoxius*, tergites **17** *Scirtothrips dorsalis*, tergites **18** *Organothrips indicus*, head & pronotum.

**Figures 19–25.**
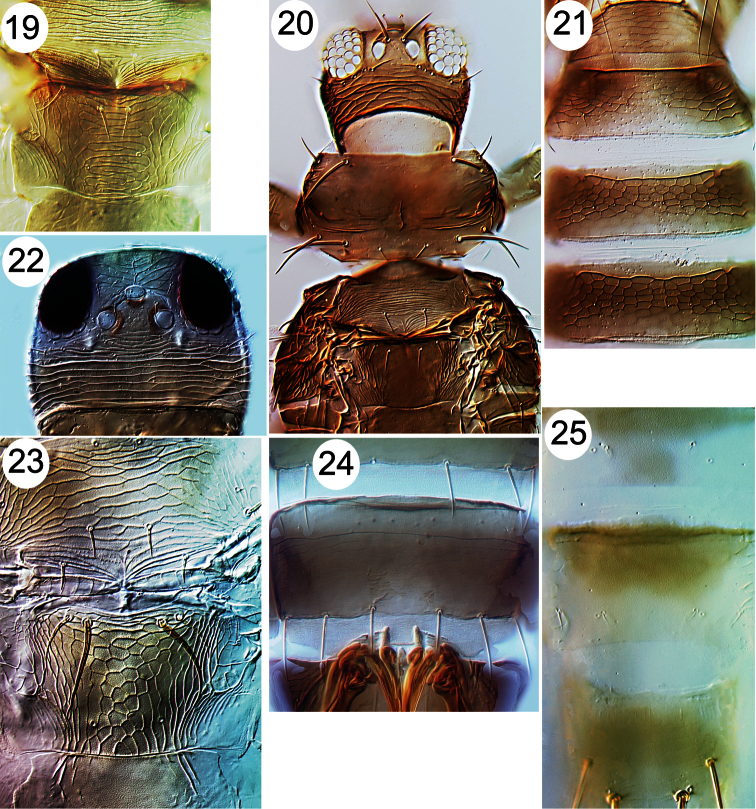
Thripinae from Java. **19** *Dichromothrips corbetti*, metanotum **20** *Ayyaria chaetophora*, head & thorax **21** *Ayyaria chaetophora*, tergites **22** *Lefroyothrips lefroyi*, head **23** *Lefroyothrips lefroyi*, metanotum **24** *Megalurothrips typicus*, sternite VII **25** *Bathrips melanicornis*, tergites VII–IX.

**Figures 26–34.**
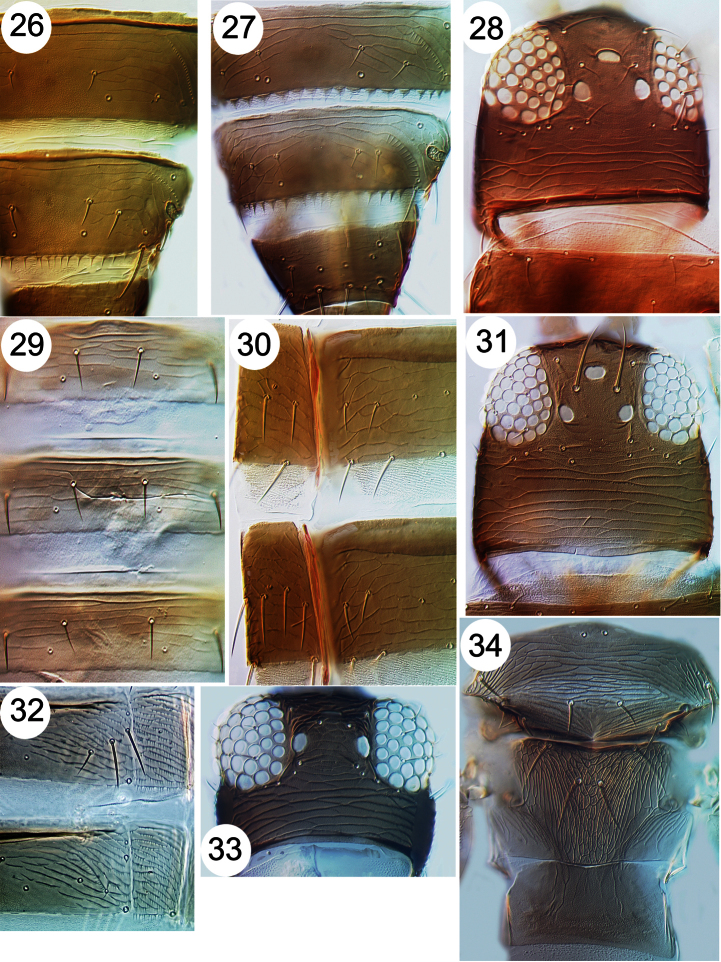
Thripinae from Java. **26** *Frankliniella intonsa*, tergites VII & VIII **27** *Microcephalothrips abdominalis* tergites VII–IX **28** *Stenchaetothrips biformis*, head **29** *Thrips nigropilosus*, tergites **30** *Thrips alliorum*, pleurotergite & sternite **31** *Thrips alliorum*, head **32** *Thrips tabaci*, tergites & pleurotergites **33** *Thrips simplex*, head **34** *Thrips simplex*, metanotum.

**Figures 35–39.**
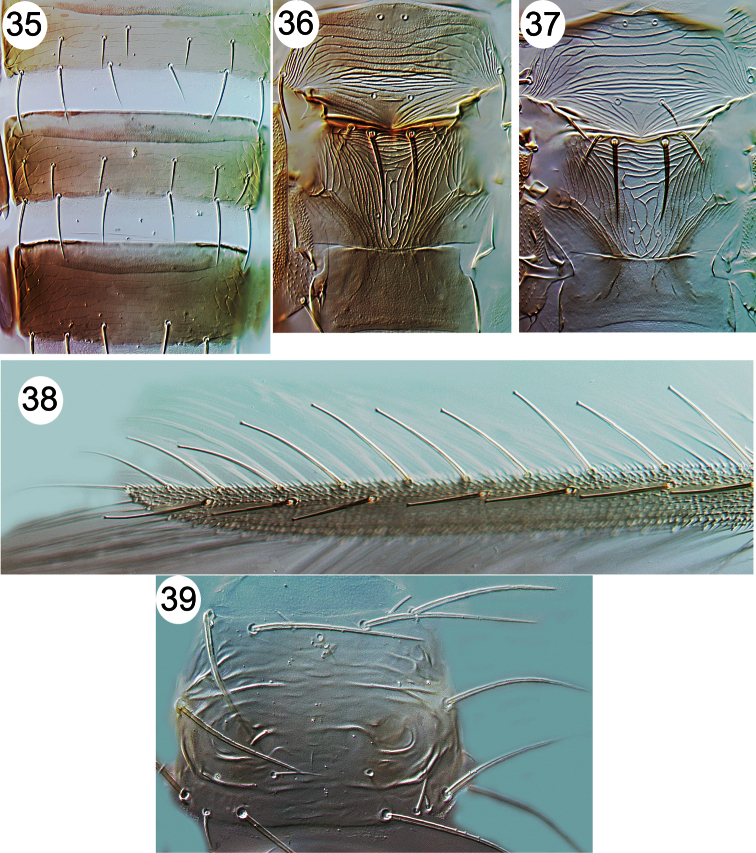
Thripinae from Java. **35** *Thrips parvispinus*, sternites V–VII **36** *Thrips florum*, meso & metanota **37** *Thrips hawaiiensis*, meso & metanota **38** *Echinothrips americanus*, fore wing **39** *Scolothrips rhagebianus*, pronotum.

### Aeolothripidae

Most of the 190 species listed in this family are from the temperate parts of the world, but a few small genera are found only in tropical countries. All the species have nine antennal segments, the sensoria on the third and fourth antennal segments form longitudinal sensory areas not emergent sense cones, and the fore wings have a broadly rounded apex and well marked veins.

** *Franklinothrips vespiformis* (Crawford DL): This predatory species was collected on the leaves of *Jatropha* at Malang in association with populations of *Zaniothrips ricini*. Originally from Central America where it is widespread, it is recorded from Thailand, Japan, New Caledonia and Australia (Mound and Reynaud 2005).

* *Mymarothrips bicolor* zur Strassen: Described from Krakatau Island, Indonesia, this species has been collected at Bogor and at Semarang, and is also known from northern Australia. It is presumably predatory and, as the name implies, is similar in appearance to one of the small parasitic Hymenoptera with the fore wings narrowed at the base but broadly rounded toward the apex.

### Thripidae

This family comprises about 2000 described species, and is found around the world wherever plants are growing. Most species have either seven or eight antennal segments, and the sensoria on the third and fourth segments are emergent and either forked or simple. The fore wings are generally slender, and commonly bear two rows of setae. Four subfamilies are recognised, although only three are recorded here.

### Thripidae – Panchaetothripinae

This subfamily comprises about 140 species in 40 genera. These are distributed throughout tropical countries, and they feed and breed on leaves not in flowers. With the exception of *Zaniothrips ricini*, the adults are dark brown, with conspicuous reticulate sculpture.

*Astrothrips* sp.: This genus comprises 12 leaf-feeding species that are found in tropical areas between Africa and northern Australia. All of them have a characteristic group of strongly recurved spines anterolaterally on the second abdominal tergite. An apparently undescribed species was collected recently at Tegal.

**Copidothrips octarticulatus (Schmutz): Only one species is recognised in this genus, and this has the second abdominal similar in structure and sculpture to species of *Astrothrips*. Presumably originally from southeast Asia, and collected at Bogor, it is also recorded from the Carribean.

*Elixothrips brevisetis* (Bagnall): Only one species is placed in this genus, and this has been found at Bogor. It is very similar in structure and biology to species of *Astrothrips*, and is widely distributed around the tropics.

**Helionothrips ananthakrishnani Wilson: Described from the eastern highlands of New Guinea on banana leaves (Wilson 1975), this species is here newly recorded from Java. Both sexes have been found on banana leaves at Bogor, Cianjur and Bandung. There are nearly 30 species listed in this genus, almost all from the Asian tropics.

*Heliothrips haemorroidalis* Bouché: Found in Bogor, the Greenhouse Thrips of temperate countries is widespread in sub-tropical areas, breeding on a wide range of plants, but usually not on herbaceous plants.

**Panchaetothrips indicus Bagnall: Described from India, females of this species have been studied from Bogor and Semarang. Six species from the Old World tropics are listed in this genus, and an identification key was provided by Mound and Postle (2004).

*Rhipiphorothrips pulchellus* Morgan: This species has the abdomen almost clear yellow in contrast to the dark brown head and thorax, and is known from India and Sri Lanka to the Philippines. It is recorded from the leaves of *Ficus* and of vines, and at Situbondo was found on *Jatropha* leaves. Four other species are listed in this genus, two from Africa and two from Asia.

*Selenothrips rubrocinctus* (Giard): The red-banded cacao thrips probably came originally from South America but is now widespread in tropical countries. It has been taken from the leaves of *Jatropha* at Bogor and at Kediri.

**Zaniothrips ricini Bhatti: Although described from *Ricinus* in India, this curiously pale-bodied species has been found several times breeding on *Jatropha* leaves at Bogor.

### Thripidae – Dendrothripinae

Adults in this subfamily have the internal furca of the metathorax greatly enlarged and “lyre-shaped”, and extending to the mesothorax. These are leaf-feeding thrips that jump actively when disturbed. About 90 species in 10 genera are recognised, and these are found particularly in tropical and sub-tropical countries.

**Asprothrips navsariensis Tyagi: Previously known only from Gujarat, in western India, living on banana leaves, both sexes have been studied from banana leaves at Bogor. The genus includes only four species, all from Asia but with one species widespread in greenhouses.

*Pseudodendrothrips* sp.: There are 19 species placed in this genus, mainly from the Old World tropics. They are minute in size, and difficult to identify to species. One species is widespread as a minor pest on the leaves of *Morus* trees used for silk-worm cultivation. A single female representing this genus was collected from *Nephelium* at Bogor.

### Thripidae – Thripinae

This is one of the two largest groups of Thysanoptera, with over 1600 recognised species in almost 250 genera. These thrips are found all over the world, many are associated with flowers, but a particularly large number of species breed only on grasses. This subfamily includes the major pest thrips, including all the tospovirus vector species.

*Anaphothrips sudanensis* Trybom: This strikingly bicoloured species was collected at Bogor. It is widespread in sub-tropical areas on various grasses, and is sometimes considered a minor pest of cereal crops. As in all 80 species of the genus *Anaphothrips*, the pronotum does not have any long setae (Mound and Masumoto 2009).

*Ayyaria chaetophora* Karny: The only species in this genus is widespread from India to Japan and Australia. It appears to breed on a wide variety of plants, including *Glycine*, *Tagetes* and *Ricinus*, and at Purwakata adults have been found on many different plant species.

*Bathrips melanicornis* (Shumsher): This species with curiously dark antennae is widespread from India across Southeast Asia to northern Australia. It has been collected at Bogor and Cianjur, and although often reported from crops, there is no reliable information concerning the plants on which it breeds. The only other species in the genus was described from India.

*Bolacothrips striatopennatus* (Schmutz): Described from Sri Lanka, but widespread across Asia to Japan, Guam and Australia, this yellow species breeds on grasses, and has been found at Cianjur. Although closely related to *Thrips* genus, the sensoria on the third and fourth antennal segments are simple not forked (Mound 2011b).

**Ceratothripoides brunneus Bagnall: Although described from West Africa, this species is common in Peninsular Malaysia, also in Thailand where it is reported as a tospovirus vector on tomatoes (Mound and Nickle 2009). A few specimens were taken at Cianjur. Although similar in appearance to *Megalurothrips* species, the eighth abdominal tergite bears a complete comb of long microtrichia.

*Craspedothrips minor (Bagnall): This is the most widespread of the 10 species in this genus, all from the Old World tropics. It is thought to be associated with the flowers of *Cassia* species (Mound et al. 2012), and at Bogor was found on *Mimosa* flowers. The posterior margins of the abdominal tergites and sternites bear a distinctive craspedum, or lobed fringe.

*Dendrothripoides innoxius* (Karny): One of only five species in this genus, this is associated with the leaves of *Ipomoea* species including sweet potato. Originally from Southeast Asia, and found at Bogor, it is also recorded from Brazil. The abdominal tergites laterally bear distinctively large, broadly based, microtrichia.

*Dichromothrips corbetti* (Priesner): Species in this genus breed on Orchidaceae in the Old World tropics, but *corbetti* has been widely distributed in greenhouses, and was recently found at Malang. Unlike the other 17 species in the genus, *corbetti* has no long setae on the pronotum.

**Echinothrips americanus Morgan: The Poinsettia Thrips is considered a pest of *Capsicum* crops under glass in Europe. It is usually associated with the leaves of various decorative Araceae and Euphorbiaceae, but is highly polyphagous. Originally from eastern North America, it has been reported recently from Cianjur in Java, also northern Australia and China (Mound et al. 2013).

**Frankliniella intonsa (Trybom): This species is widespread from Europe to Taiwan, and has been found at Cianjur and Bandung on rose and on strawberry plants. It is similar in structure and biology to the major pest species, *Frankliniella occidentalis*, from which it is distinguished by the short setae behind the eyes and the absence of campaniform sensilla on the metanotum.

*Frankliniella schultzei* (Trybom): Presumably originally from South America, this species is widespread throughout tropical countries. A tospovirus vector, it is distinguished from most species of *Frankliniella* by the lack of a marginal comb on the eighth tergite of females, and the presence of a pair of setae between the posterior pair of ocelli. It appears to be variable in colour, from yellow to brown, and a single brown male was collected at Bogor.

*Lefroyothrips lefroyi* (Bagnall): Only four species are listed in this genus, of which this one is associated with the flowers of tea plants in northern India. A single female has been seen from Cianjur.

*Megalurothrips distalis* (Karny): Species of this genus breed in the flowers of Fabaceae in the Old World tropics. Although 13 species are described, they cannot all be recognised (Palmer 1987). Females of *distalis* are distinguished from the more common *usitatus* by the dark brown colour of the third antennal segment. This species was found at Cianjur.

*Megalurothrips typicus* Bagnall: This species can be distinguished by the presence of all three pairs of setae arising at the posterior margin of the seventh sternite in females, instead of the median pair arising sub-marginally. Also, the antennae are more extensively yellow than in the other members of the genus. It has been found at Tegal.

*Megalurothrips usitatus* (Bagnall): This is the most common thrips in the flowers of cultivated legume plants across most of tropical Asia (Palmer 1987), and has been seen from Bogor and Tegal.

*Microcephalothrips abdominalis* (Crawford DL): Common in the flowers of many Asteraceae, this sub-tropical species is often associated with crops of *Helianthus*. Found at Bogor, Bandung and Cianjur, it is similar to species of genus *Thrips* in having ctenidia laterally on the tergites, but the posterior margin of the tergites bears a distinctive row of triangular teeth.

** *Mycterothrips* sp. This genus is widespread around the world, with the species apparently all leaf-feeding, and one a pest in Asia on *Glycine max*. One female of a bicoloured species has been seen from Banjarnegara, a highland area in central Java. This apparently undescribed species has discal setae on the sternites, a character state that is found in only five of the 29 species described in this genus.

** *Organothrips indicus* Bhatti: This species breeds underwater in the mucilage on the stems of some aquatic plants, such as *Eichornia crassipes*, and *Typha* species. It is recorded from India and also from northern Australia, and was found at Demak. A closely similar species is recorded from the Pacific islands (Mound 2000).

*Rhamphothrips* sp.: The females of species in this genus are particularly difficult to identify. However, specimens taken at Bogor were readily recognised as members of this genus, because the median two pairs of marginal setae on the seventh sternite arise very close together in the mid-line (Mound and Tree 2011).

*Scirtothrips dorsalis* Hood: Species of this genus have closely spaced transverse rows of microtrichia laterally on the abdominal tergites. This Oriental species is a widespread pest on several crops. It was collected at Bogor, and is a tospovirus vector in India, and introduced to Israel and the Caribbean. It is unusual amongst the 100 described species of *Scirtothrips* in having microtrichia almost covering all of the sternites, instead of these being restricted to the lateral areas (Mound and Stiller 2011).

**Scolothrips rhagebianus Priesner: Very similar to the North American *Scolothrips sexmaculatus*, this species is recorded from Egypt to Australia, and has been seen from Malang. All *Scolothrips* species have six pairs of long setae on the pronotum, and are predators of spider mites (Mound 2011a).

*Stenchaetothrips biformis* (Bagnall): The rice thrips is widespread across Asia, on grasses as well as rice, and has been seen from Bogor and Cianjur. *Stenchaetothrips* species are very similar to *Thrips*, but have the lateral pair of setae between the eyes longer than the median pair (Ng and Mound 2012).

**Thrips alliorum (Priesner): Apparently breeding on onions, this species is recorded widely in southeast Asia, and also in Hawaii, and has been seen from Cianjur. It is dark brown, with the head projecting slightly in front of the eyes.

*Thrips coloratus* Schmutz: This species varies greatly in colour, from pale yellow to brown, but the most common form is brown medially on the tergites, and tergite ten is also brown. It is a flower-living species that is known widely across Southeast Asia into northern Australia, and was collected at Bogor.

*Thrips florum* Schmutz: This is one of the most common and widespread flower thrips across Asia to the Pacific islands, and is also introduced to the Caribbean and southern Florida. Collected at Bogor, and at Cianjur, it is very similar to *hawaiiensis* in structure and biology (Mound and Masumoto 2005).

*Thrips hawaiiensis* (Morgan): This is the most commonly collected flower thrips across Asia to the Pacific and eastern Australia, and was found at both Bogor and Cianjur. It is also introduced to the southern parts of the USA, as well as some Caribbean islands.

*Thrips javanicus* Priesner: Specimens identified as this species were taken at Bogor. The species requires further study as it is not clearly distinguished from several other Asian species that lack sternal discal setae (Mound 2005).

*Thrips malloti* Priesner: Widespread from India to northern Australia, this species was collected at Bogor. The fore wing is dark except at the base, and the first vein has an almost complete row of setae, in contrast to *javanicus*.

*Thrips melastomae* Priesner: This is another poorly defined species that is recorded from Malaysia to the Philippines and was taken at Bogor. Palmer (1992) suggests that it may be associated with species of the plant genus *Melastoma*.

*Thrips nigropilosus Uzel: This polyphagous pest species has been found at Cianjur infesting the leaves of carrots, but in Europe it is associated with various other crops including lettuce, and in Kenya has been reported as a pest of Pyrethrum crops. The wings vary from fully complete to shorter than the thoracic width, and the median setae on the tergites are unusually long.

*Thrips palmi* Karny: A major pest species and tospovirus vector, this species is sometimes abundant in southeast Asian countries causing severe leaf damage to various crops such as egg plant. It has been taken from various crop plants in Java at Bandung, Bogor and Cianjur.

*Thrips parvispinus* (Karny): In Indonesia, including Java, this is probably the most widespread and commonly collected thrips species (Mound and Collins 2000). The body size and colour are variable, probably in relation to the temperature during development. The body and wing lengths were greater on specimens from *Capsicum* in the highlands of West Java than in specimens from this crop in the lowlands (Sartiami et al. 2011). At large populations this species is likely to damage crops such as capsicum and potato, and it has been take at Bogor, Cianjur and Bandung.

*Thrips simplex* (Morison): The Gladiolus Thrips is found throughout the world wherever these flowers are grown. Feeding by this thrips causes the flowers and leaves to be marked by pale streaks, and specimens have been studied from Bogor.

**Thrips safrus Mound & Masumoto: Collected recently at Bogor, this is the first record of this polyphagous species from outside Australia, where it is common in the northern tropical and sub-tropical parts of the continent (Mound and Masumoto 2005).

*Thrips tabaci* Lindeman: One of the most widespread species of thrips, the Onion Thrips does not usually occur in the wet tropics. It can produce large populations on various crops under hot dry conditions, and has been found at Cianjur.

**Thrips unispinus Moulton: described from New Guinea, large numbers of this species have been found in Mango flowers in northern Australia, and it is here recorded from Cianjur.

**Table 1.** Plants from which thrips have been collected in Java. <br/>

**Family Name**	**Host Plant**	**Thrips species**
Acanthaceae	*Ruellia* sp.	*Copidothrips octarticulatus*<br/> *Thrips parvispinus*
Amaryllidaceae	*Allium* spp.	*Bolacothrips striatopennatus*<br/> *Thrips alliorum*
Anacardiaceae	*Mangifera indica*	*Thrips hawaiensis*
Annonaceae	*Polyalthia longifolia*	*Helionothrips ananthakrishnani*
Apiaceae	*Coriander sativum*	*Thrips parvispinus*
	*Daucus carota*	*Thrips nigropilosus*<br/> *Thrips parvispinus*<br/> *Thrips tabaci*<br/> *Megalurothrips typicus*<br/> *Megalurothrips usitatus*
Apocynaceae	*Allamanda cathartica*	*Thrips nigropilosus*<br/> *Thrips malloti*
Araceae	*Colocasia* sp.	*Heliothrips haemorrhoidalis*
Arecaceae	*Elais guineensis*	*Thrips hawaiiensis*<br/> *Thrips melastomae*
Asteraceae	*Lactuca sativa*	*Thrips parvispinus*
	*Ageratum* sp.	*Microcephalothrips abdominalis*
Brassicaceae	*Brassica oleracea*	*Thrips nigropilosus*
	*Brassica rapa*	*Thrips parvispinus*
Burseraceae	*Canarium sp*.	*Helionothrips ananthakrishnani*
Caricaceae	*Carica papaya*	*Thrips hawaiiensis*<br/> *Thrips parvispinus*
Clusiaceae	*Garcinia mangostana*	*Scirtothrips dorsalis*<br/> *Thrips hawaiiensis*
Convolvulaceae	*Ipomoea aquatica*	*Thrips parvispinus*
	*Ipomoea batatas*	*Mymarothrips bicolor*<br/> *Bathrips melanicornis<br/> Microcephalothrips abdominalis*
Cucurbitaceae	*Cucumis sativus*	*Organothrips indicus*<br/> *Thrips palmi<br/> Thrips parvispinus*
	*Momordica charantica*	*Megalurothrips usitatus*
	*Luffa acutangula*	*Thrips parvispinus*
Cyperacea	*Cyperus*	*Thrips nigropilosus*
Euphorbiaceae	*Aleurites mollucana*	*Helionothrips ananthakrishnani*
	*Jatropha curcas*	*Franklinothrips vespiformis*<br/> *Mymarothrips bicolor*<br/> *Heliothrips haemorrhoidalis*<br/> *Rhipiphorothrips pulchellus*<br/> *Selenothrips rubrocintus*<br/> *Zaniothrips ricini*<br/> *Scolothrips rhagebianus*
Fabaceae	*Mimosa pudica*	*Thrips parvispinus*<br/> *Thrips malloti*<br/> *Crapedothrips minor*
	*Vigna unguiculata*	*Thrips hawaiiensis*<br/> *Thrips parvispinus*<br/> *Megalurothrips usitatus*
	*Phaseolus vulgaris*	*Thrips parvispinus*
	*Cicer arietinum*	*Bathrips melanicornis*<br/> *Megalurothrips usitatus*
	*Arachis hypogaea*	*Megalurothrips usitatus*
Iridaceae	*Gladiolus* sp.	*Thrips simplex*
Lamiacea	*Ocimum* sp.	*Astrothrips* sp.<br/> *Bathrips melanicornis*<br/> *Frankliniella schultzei*<br/> *Thrips palmi*
Lauraceae	*Persea americana*	*Thrips hawaiiensis*
Melastomataceae	*Melastoma* sp.	*Bathrips melanicornis*<br/> *Rhamphothrips* sp.
Musaceae	*Musa* spp.	*Asprothrips navsariensis*<br/> *Helionothrips ananthakrishnani*
Myrtaceae	*Psidium guajava*	*Thrips hawaiiensis*
	*Syzygium* sp.	*Scirtothrips dorsalis*
Orchidaceae	*Dendrobium* sp.	*Dichromothrips corbetti*<br/> *Thrips parvispinus*
Oxalidaceae	*Averrhoa carambola*	*Asprothrips navsariensis*<br/> *Heliothrips haemorrhoidalis*<br/> *Thrips javanicus*
Pandanaceae	*Pandanus* sp.	*Heliothrips haemorrhoidalis*<br/> *Elixothrips brevisetis*
Phyllanthaceae	*Sauropus androgynus*	*Bathrips melanicornis*<br/> *Echinothrips americanus*<br/> *Thrips palmi*<br/> *Thrips parvispinus*
Poaceae	*Imperata* sp.	*Megalurothrips usitatus*
	*Zea mays*	*Thrips hawaiiensis*<br/> *Thrips nigropilosus*<br/> *Thrips parvispinus*
	*Oryza sativa*	*Selenothrips rubrocinctus*<br/> *Microcephalothrips abdominalis*
	*Triticum aestivum*	*Anaphothrips sudanensis*
	*Axonopus* sp.	*Bathrips melanicornis*
Rosaceae	*Rosa* spp.	*Bathrips melanicornis*<br/> *Bolacothrips striatopennatus*<br/> *Ceratothripoides brunneus*<br/> *Frankliniella intonsa*<br/> *Microcephalothrips abdominalis*<br/> *Thrips coloratus*<br/> *Thrips florum*<br/> *Thrips parvispinus*<br/> *Thrips unispinus*<br/> *Megalurothips distalis*<br/> *Megalurothrip usitatus*<br/> *Lefroyothrips lefroyi*
	*Fragaria vesca*	*Frankliniella intonsa*<br/> *Thrips palmi*
Rutaceae	*Citrus* spp.	*Heliothrips haemorrhoidalis*<br/> *Thrips hawaiiensis*<br/> *Thrips malloti*
Sapindaceae	*Nephelium lappaceum*	*Pseudodendrothrips* sp.<br/> *Thrips coloratus*<br/> *Thrips parvispinus*
Solanaceae	*Nicotiana tabacum*	*Thrips parvispinus*
	*Solanum lycopersicum*	*Thrips palmi*<br/> *Thrips parvispinus*
	*Solanum tuberosum*	*Thrips parvispinus*
	*Solanum nigrum*	*Thrips parvispinus*
	*Solanum melongena*	*Thrips palmi*<br/> *Thrips parvispinus*
	*Datura metel*	*Thrips palmi*
	*Capsicum annuum*	*Bathrips melanicornis*<br/> *Microcephalothrips abdominalis*<br/> *Thrips hawaiiensis*<br/> *Thrips parvispinus*
Verbenaceae	*Lantana camara*	*Dendrothripoides innoxius*<br/> *Bathrips melanicornis*<br/> *Thrips florum*<br/> *Thrips malloti*
Zingiberaceae	*Curcuma longa*	*Panchaetothrips indicus*
	*Curcuma zantorrhiza*	*Panchaetothrips indicus*
